# Bone Effects of Biologic Drugs in Rheumatoid Arthritis

**DOI:** 10.1155/2013/945945

**Published:** 2013-06-20

**Authors:** Addolorata Corrado, Anna Neve, Nicola Maruotti, Francesco Paolo Cantatore

**Affiliations:** Rheumatology Clinic, Department of Medical and Surgical Sciences, University of Foggia, Ospedale “Col. D'Avanzo”, Viale degli Aviatori, 71100 Foggia, Italy

## Abstract

Biologic agents used in the treatment of rheumatoid arthritis (RA) are able to reduce both disease activity and radiographic progression of joint disease. These drugs are directed against several proinflammatory cytokines (TNF**α**, IL-6, and IL-1) which are involved both in the pathogenesis of chronic inflammation and progression of joint structural damage and in systemic and local bone loss typically observed in RA. However, the role of biologic drugs in preventing bone loss in clinical practice has not yet clearly assessed. Many clinical studies showed a trend to a positive effect of biologic agents in preventing systemic bone loss observed in RA. Although the suppression of inflammation is the main goal in the treatment of RA and the anti-inflammatory effects of biologic drugs exert a positive effect on bone metabolism, the exact relationship between the prevention of bone loss and control of inflammation has not been clearly established, and if the available biologic drugs against TNF**α**, IL-1, and IL-6 can exert their effect on systemic and local bone loss also through a direct mechanism on bone cell metabolism is still to be clearly defined.

## 1. Introduction

Chronic inflammatory diseases are frequently associated with systemic bone loss, whose pathogenesis is extremely complex and involves different mechanisms that are strictly interrelated. The relationship between inflammation and bone loss has been clearly established in many clinical and experimental models [1–4]. Particularly, many studies have focused on systemic bone loss that occurs in various chronic inflammatory diseases currently observed in clinical practice, such as inflammatory bowel diseases, chronic lung inflammation, vasculitis, connective tissue diseases, and inflammatory joint diseases [5–7]. In these diseases, the physiopathological mechanisms underlying the systemic bone loss are partly shared, but are in part distinct from each other, and specific treatment for these pathological conditions may also affect bone loss in different ways [8]. The causes of bone loss in chronic inflammatory diseases are multiple, and various experimental models together with clinical evidence suggest that a major role is played by proinflammatory cytokines, such as TNF*α* [9].

Rheumatoid arthritis (RA) represents one of the most typical examples of systemic inflammatory processes which lead to significant changes in bone metabolism. The bone involvement typically observed in RA is represented by generalised osteoporosis and localised bone loss, the latter including erosions and juxta-articular osteopenia of affected joints. Many epidemiological studies clearly established the presence of generalized bone loss; RA patients present lower hip and vertebral bone mineral density (BMD) and a higher fracture risk compared to age and gender matched controls. Disease activity is related to generalized bone loss and low BMD; an accelerated BMD loss at spine and hip is observed in the early stages of RA compared to controls [2], and in early RA vertebral fracture can occur in the first year of the disease irrespective of the cumulative prednisone dose. The causes of generalised osteoporosis in RA are various and include disease activity, immobility, and corticosteroid use [10], whereas the main cause of both periarticular osteopenia and local erosions is represented by the chronic inflammation of synovial membrane, which presents a strict interaction with the juxta-articular bone. However, recent scientific data suggests that these three type of bone loss are at least in part mediated by common pathogenic mechanisms [11], that converge especially toward an alteration of bone remodelling processes characterised by the increase of osteoclast activity, with a negative balance of bone formation and resorption. Several studies suggest that inflammation itself plays an essential role in bone loss; in the recent years, many key interactions between inflammation and bone have been revealed. Particularly, it has been shown that various mediators expressed within the synovial tissues are potentially able to modify the bone remodelling processes promoting bone resorption. Thus, the control of inflammation appears to be one of the most important strategies for prevention of bone loss in RA [11]. 

## 2. Relationship between Joint Inflammation and Bone Loss in RA

A great number of local and systemic factors can control bone remodeling by acting on osteoclasts and osteoblasts. Some cytokines, including IL-1, TNF*α*, IL-6, IL-11, and IL-17, are able to act both directly on osteoblasts, exerting on these cells different effects, those on osteoclasts and osteoclast progenitors, stimulating osteoclastogenesis and regulating osteoclast activity. The main proinflammatory cytokines IL-1, IL-6, and TNF*α* are found in higher concentrations in the synovial fluid and tissues of RA patients and represent the key mediators implicated in inflammatory and immune responses underlying the pathogenesis of this disease. All of these cytokines are able to negatively affect bone metabolism with different mechanisms and consequently are involved in the pathogenesis of both generalised and local bone loss. Macrophages represent the main source of inflammatory cytokines and the number of macrophages present at the bone-synovial interface correlate with the degree of the bone damage.

The majority of pathogenic mechanisms involved in systemic and local bone loss in RA converge to the increase of osteoclastogenesis and osteoclast activity. Osteoclast activation greatly depends on stimulation exerted by the receptor activator of nuclear factor *κ*β ligand (RANKL) which binds to the receptor activator of nuclear factor (RANK) on osteoclast surface. RANKL, a protein belonging to the TNF*α* superfamily, and its inhibitor osteoprotegerin (OPG) are crucial for bone physiology and inflammation [12], as the expression of RANKL is stimulated by proinflammatory cytokines (TNF*α*, IL-1, IL-6, and IL-17). The regulation of NF-*κ*β pathway by the RANK/RANKL/OPG system is essential for osteoclastogenesis and osteoclast activity, but NF-*κ*β signalling can be activated also by TNF*α* via TNF receptor 1 (TNFR1) [13], which is expressed on osteoclast precursors. Other than in macrophages of inflamed synovium, TNF*α* is produced in a large amount in RA by osteoblasts and a wide range of inflammatory cells, including lymphocytes and fibroblasts [14]. TNF*α* promotes bone resorption in RA, as it is able to increase osteoclast recruitment, differentiation, and activity both directly, in the presence of minimal concentration of RANKL or even in the absence of RANKL signalling [15, 16] and indirectly by increasing the expression of osteoclast activators (M-CSF and RANKL) in several cells such as osteoblasts and cells of immune system [17–19]. Its negative effect on bone metabolism makes TNF*α* an ideal candidate for linking inflammation and bone loss. 

An increase in bone resorption processes should be associated to a concomitant increase in bone formation due to the strength coupling of bone formation and resorption, but in RA bone resorption is unbalanced by an appropriate bone formation, which is inadequate or even suppressed. It can be supposed that some proinflammatory cytokines are also able to suppress bone formation; particularly it has been shown that TNF*α* can inhibit osteoblast differentiation [20]. Indeed, although the increase of bone resorption represents the main mechanism involved in inflammation-related bone loss, it has been shown from in vitro studies that TNF*α* can also increase osteoblast apoptosis [21] and reduce osteoblast differentiation and proliferation, still through TNFR1 receptor. Recent data showed that the inhibition of osteoblast differentiation by TNF*α* is mediated by the reduction of RUNX2 and Osterix expression, which are essential regulators in various stages of osteoblast differentiation [22, 23]. It has also been shown that TNF*α* may suppress osteoblast-mediated bone formation by the inhibition of Wnt-β-catenin pathway, which is one of the main bone forming canonical ways, through the upregulation of the Wnt inhibitors Dickkopf-related protein (Dkk-1) and sclerostin [24].

Other than TNF*α*, a wide variety of inflammatory cytokines affect bone remodelling in patients with RA, both indirectly by modulating RANK/RANKL system and through a direct effect on osteoclastogenesis [25–29]. T lymphocytes are an essential source of many cytokines that exert a stimulatory effect on osteoclastogenesis, such as IL-1β, IL-6, and IL-11, and it can be hypothesized that in inflammatory chronic diseases there is a T-cell-mediated osteoclastogenesis. 

Many clinical and experimental data support the crucial role of IL-1 in RA, which is considered among the master cytokines in these disease. Transgenic mice deficient in IL-1 receptor antagonist (IL-1Ra) spontaneously develop a chronic arthritis similar to RA with bone erosions and present higher susceptibility for collagen-induced arthritis [30]. Conversely, in an experimental animal model of streptococcal cell-wall induced arthritis, IL-1-deficient mice show a diminished tissue damage and synovial infiltrate, without any reduction in joint swelling, suggesting that IL-1 might be involved in joint damage whereas inflammation is regulated by other additional mechanisms. In the same experimental model of chronic arthritis, blocking of IL-1 resulted in little or absent suppression of inflammation, but induced a normalization of chondrocyte activity, confirming that IL-1 exerts a positive effect on cartilage and bone degradation [31]. Other studies showed that IL-1 is not predominant in the acute inflammatory stages of most experimental arthritis models, but plays a significant role in perpetuating joint inflammation and in the pathogenesis of bone and cartilage damage [32]. IL-1 is among the most potent activators of osteoclastogenesis and exerts this activity by stimulating the production of RANK-L in T cells. IL-1 increases RANKL expression also in osteoblast lineage cells and regulates the production of OPG, the natural inhibitor of RANKL. Further, IL-1 can directly increase osteoclastogenesis and synergize with RANKL in potentiating osteoclast-mediated bone resorption.

IL-6 is another pleiotropic cytokine that plays a key role in inflammation and autoimmunity processes, including those underlying the pathogenesis of RA where it acts synergistically with IL-1 and anti-TNF*α*. IL-6 levels are increased both in serum and synovial fluid of patients with RA [33, 34] and positively correlate with disease activity. By binding to its receptor (IL-6R), IL-6 exerts a broad spectrum of inflammatory events that are essential in RA. IL-6 is a major stimulator of the synthesis of acute-phase reactants, exerts a role in the recruitment of leucocytes and other inflammatory cells, and stimulates synoviocyte proliferation. IL-6 is also a key regulator of bone remodelling as it is able to induce osteoclast differentiation and activation [35], playing a crucial role in the pathogenesis of local and systemic bone loss associated with RA [36].

Based on the clinical and experimental evidence of the strong relationship between inflammation and bone loss, and considering the role played by TNF*α*, IL-1, and IL-6 in the pathogenesis of changes in bone metabolism in RA, it can be hypothesized that treatments able to inhibit chronic inflammation, and particularly biological agents directed against these cytokines, could potentially inhibit or reverse the different kinds of bone loss observed in this disease. At present, there are few data concerning the effects of treatments directed against TNF*α*, IL-1, and IL-6 on systemic bone loss in RA patients.

## 3. Treatment of RA with Biologic Drugs and Bone Loss

Other than nonsteroidal anti-inflammatory drugs and corticosteroids, traditional treatment of RA consists in disease-modifying antirheumatic drugs (DMARDs), which act as immune-modulating agents exerting their effect on the autoimmune processes underlying the pathogenesis of disease. Nevertheless, in the latest 15 years the treatment of RA has been revolutionised by the development of agents that target a specific molecular mechanism of the inflammatory cascade, the so-called biologic drugs. Inhibitors of TNF*α*, such as Infliximab, Adalimumab, Certolizumab, and Golimumab, are the most commonly used biologic therapies in RA, but agents directed against other proinflammatory cytokines involved in the pathogenesis of RA have been developed, such as anti-IL-1 (Anakinra) and anti-IL-6 (Tocilizumab). All these agents have shown to be able to reduce both disease activity and radiographic progression of joint disease. Taking into account that the same cytokines are involved both in local and generalized bone loss, it is rational to speculate that biologic agents could directly perform a protective action on bone remodelling, even if probably the different biologic drugs exert variable effects on local and systemic bone resorption typical of RA.

### 3.1. TNF*α* Blockade and Bone Loss Prevention in RA

Anti-TNF*α* agents are the first biological drugs used for treatment of RA; their effectiveness in controlling disease activity and inflammation and in preventing joint structural damage has been proved in several large randomized clinical trials. This group of drugs includes the monoclonal anti-TNF*α* antibodies (Infliximab, Adalimumab, Golimumab an Certolizumab) and the soluble TNF*α* receptor Etanercept.

It has been supposed that anti-TNF*α* therapy could be effective both in controlling chronic inflammation than in preventing or reversing systemic osteoporosis and local bone loss (erosion and juxta-articular osteoporosis) typically observed in RA and that its positive effect on bone could be independent of anti-inflammatory properties.

The potential positive effect of anti-TNF*α* on bone loss in RA patients has been shown in various experimental models. In transgenic mice overexpressing TNF*α*, which develop a destructive arthritis closely mimicking human RA, TNF*α* blockade completely reversed the increased bone resorption and led to a dramatic increase in osteoblast numbers, with a positive net balance of bone turnover [37]. In an experimental animal model of collagen or adjuvant induced arthritis, anti-TNF*α* and anti-IL-1 therapy inhibited systemic and local inflammation and reduced local bone loss, showing no effects on generalized bone loss; conversely, RANK-L treatment was able to prevent both local and systemic bone loss, without effects on inflammation parameters [38]. Anti-TNF*α* treatment significantly increased total body bone mineral density (BMD) in an animal model of collagen-induced arthritis, with increase in trabecular thickness and no changes in bone volume or trabecular separation, suggesting a preservation of bone formation [39].

TNF*α* blockade can act directly by preventing the direct stimulatory effect of TNF*α* on osteoclastogenesis but, based on the ability of TNF*α* to directly increase RANKL expression it has been hypothesised that TNF*α* inhibition could act through the reduction of RANKL [40]. Further, anti-TNF*α* treatment could prevent the negative effect of TNF*α* on osteoblast activity and differentiation. 

The majority of clinical studies that evaluated the effect of anti-TNF*α* agents on bone loss in RA, irrespective of their effects on joint inflammation, had primarily focused on bone turnover markers rather than on other clinically important endpoints, such as BMD and/or fracture risk. Several studies showed that anti-TNF*α* treatment induces a significant decrease in bone resorption markers [41–43], such as serum C-terminal cross-linked telopeptide of type I collagen, and enhances bone formation markers (osteocalcin and procollagen serum type I N-terminal propeptide), which represent the expression of a change in bone remodelling processes favouring a positive net bone balance [42, 44]. Further, anti-TNF-*α* agents are able to reduce circulating RANKL, resulting in a favourable change in OPG/RANKL ratio. The results of these studies, although discordant, showed a tendency toward a modest and transitory increase in bone formation and a more important decrease in bone resorption markers, thus supporting the hypothesis that TNF*α* blockade exerts a more effective action on osteoclastogenesis/osteoclast activity rather than on osteoblastogenesis/osteoblast activity.

Reports consistent with effects of TNF*α* blockade on BMD have begun to emerge in recent years [42, 43, 45]. Most studies showed a stable or even increased BMD in patients with RA treated with TNF*α* inhibitors. It has been reported that anti-TNF*α* therapy is able to inhibit bone loss at spine and hip [42–44, 46–48] even if comparative studies showed conflicting results. One-year treatment with the anti-TNF*α* Infliximab associated to Methotrexate was able to prevent spine and hip bone loss in patients with RA, compared to patients receiving only the conventional treatment with Methotrexate [49]. The protective effect on BMD was independent of sex, age, menopause status, and steroid use; further, it was observed also in patients who did not exhibit a clinical joint improvement, suggesting that the positive role of anti-TNF*α* therapy was independent of inflammation control. No changes in bone resorption/formation markers from baseline or between the groups were observed, even if a slightly greater reduction of both serum osteocalcin and carboxy-terminal telopeptide of type I collagen (CTX-I) in patients treated with Infliximab suggested a greater decrease in bone remodelling with this drug. However, in some single-arm studies with anti-TNF*α* agents, the inhibition of bone loss was accompanied by improvement in disease activity and/or reduction of inflammation [42, 47] indicating that the protective effects of TNF*α* agents were strictly related to their anti-inflammatory activity rather than a direct and independent effect on bone.

The use of Infliximab over 2 years in patients with RA induced a significant increase in BMD at lumbar spine [50]. Another open-label, prospective study showed that the anti-TNF*α* monoclonal antibody Adalimumab preserved the bone loss at spine and femoral neck in RA patients treated for 1 year [47], confirming the stop of bone loss after TNF*α* blockade. Although generalised and local bone loss in RA share many physiopathological mechanisms, some clinical studies showed a dissociation in the antiresorptive effect of anti-TNF*α* agents between hands and hip or spine, suggesting that periarticular bone of hands is more sensitive to the local effect of proinflammatory cytokines released by the adjacent synovial tissue [42, 45, 50]. 

It has been reported that Infliximab associated to Methotrexate reduced BMD loss at hip compared to treatment with Methotrexate alone, but this effect was not observed at lumbar spine and hands [45]. A large randomized clinical trial [51], in which the differences on bone loss between traditional disease-modifying antirheumatic drugs and various anti-TNF*α* regimens were evaluated, showed that conventional treatment alone was associated with a greater hand bone loss compared to the association with anti-TNF*α* therapy; however, these effects disappeared when results were adjusted for disease activity. Further, a post hoc analysis grouping patients by therapeutic response showed that the protective effect on bone was associated with clinical remission, irrespective of treatment with anti-TNF*α* [52]. Conversely, a second well-powered randomized clinical trial showed that the anti-TNF*α* Adalimumab in combination with Methotrexate reduced hand bone loss independently of clinically assessed disease activity and inflammatory status [53], suggesting that the beneficial effects of anti-TNF*α* therapy could not be limited to the control of inflammation, but also to its ability to inhibit the direct effect of TNF*α* on osteoclast activation by binding to TNF*α* receptor placed on osteoclast precursors.

These data support the hypothesis that the treatment of the underlying chronic inflammation is not the predominant mechanism of the beneficial effects of anti-TNF*α* agents on bone. It is also possible that the beneficial effect on BMD can be due to decreased pain, increased physical activity, or improved nutritional status, other than to a direct effect on bone cells.

The effect of TNF*α* on fracture risk remains uncertain, as changes in BMD and serum bone remodeling markers can be useful in predicting the risk of osteoporotic fracture, but many other factors, including trabecular microarchitecture, may also influence this outcome [54]. However, a recent population-based cohort study [55] showed that the risk of nonvertebral fractures did not differ between patients with RA receiving TNF*α* inhibitors with or without a nonbiologic DMARD and those receiving a nonbiologic DMARD alone.

### 3.2. IL-1 and IL-6 Blockade and Bone Loss Prevention in RA

Tocilizumab, a humanized anti-IL-6 monoclonal antibody, is the only approved biological drug targeted against IL-6; it acts by binding to the two forms of IL-6 receptor (IL6R) and prevents the formation of IL-6/IL6R complexes. The positive effects of anti-IL-6 on control of chronic inflammation and on prevention of the structural joint damage and improvement of physical function [56] in RA patients have been proven by various large randomized clinical trials, but the effects on generalized bone loss have not yet fully investigated. In a recent randomized, double-blind, placebo control clinical trial, performed on anti-TNF*α* refractory RA patients [36] it has been shown that after 16 weeks of treatment Tocilizumab determined a strong and significant decrease of the circulating levels of bone resorption markers (CTX-I), whereas it did not induce any significant changes in the bone formation markers osteocalcin and propeptide of type I collagen (PINP), reflecting a net positive effect on bone balance. Interestingly, the positive correlation between inflammation (CRP) and disease activity (DAS 28) with bone resorption markers found before anti-IL-6 treatment was lost after 16 weeks of anti-IL-6 exposure, suggesting that this therapy could interfere with the interaction between systemic inflammation and bone resorption in RA. However, the therapeutic inhibition of IL-6 receptor in patients with RA can affect bone homeostasis through an effect on mechanisms that control bone formation. It has been reported that a short course of IL-6 inhibition in patients with RA induced changes in serum levels of the natural inhibitors of the canonical Wnt signalling. Particularly, after two monthly infusion of Tocilizumab, Dkk-1 circulating levels were reduced; conversely, sclerostin levels were increased, probably due to a balance effect related to the reduced osteoclast function and/or to the reduction of Dkk-1. The observed changes in serum levels of Dkk-1 and sclerostin were comparable between patients who achieved remission or low disease activity after Tocilizumab treatment and those did not, confirming the hypothesis that IL-6 blockade can exert an influence on bone metabolism irrespective of its effect on suppression of systemic inflammation through a role in the regulation of Wnt pathway. The effects of IL-6 inhibition on bone in patients with RA have been reported also in a phase II randomized multicenter double-blind placebo-controlled trial in which the effect on bone turnover markers of two different dose regimens of Tocilizumab were evaluated [57]. This study reported an early and sustained increase in circulating levels of bone formation marker PINP with both Tocilizumab dose regimens and an increased osteocalcin level with the higher dose; conversely, the bone resorption markers CTXI and ICTP were significantly decreased. These data confirm the hypothesis that IL-6 inhibition can induce a beneficial effect on bone turnover and could be able to reverse the negative bone balance observed in RA patients.

Nevertheless, data on the possible effect of IL-6 inhibition on BMD and fracture risk remain to be determined, and further studies are required to clearly establish the real beneficial effect of IL-6 inhibition on the prevention of systemic and local bone loss in RA and if the potential beneficial effects are related to the reduction of inflammation status or are due to the direct effect on bone metabolism.

The effect of IL-1 blockade on bone resorption had been previously evaluated in different experimental animal models. In a model of collagen-induced arthritis, treatment with anti-IL-1 antibodies was associated with a significant reduction in clinical score, the prevention of cellular infiltration and cartilage damage and with the abolition of bone erosions [58]. In an adjuvant arthritis model in rats, treatment with IL-1Ra induced a significant reduction of bone resorption compared to controls, and this antiresorptive effect was associated with a significant reduction in the number of osteoclasts [59].

 Anakinra is a recombinant IL-1 receptor antagonist (IL-Ra) that has been approved for the treatment of RA. The positive effect of Anakinra on clinical parameters in patients with RA was demonstrated in a large randomized controlled clinical trial, in which Anakinra provided significantly greater clinical improvement than placebo [60]. Further, Anakinra significantly reduced the progression of bone erosion in treated patients compared to placebo [60]. The effect of IL-1 blockade on bone metabolism has been proven in ovariectomized rodents [61] and in postmenopausal women, in which Anakinra can partially prevent the increase of bone resorption markers due to estrogen deficiency [62]. Nevertheless, to date there are not published studies concerning the effect of Anakinra on BMD and/or fracture risk in patients with RA. 

## 4. Conclusions

The link between bone cell, inflammation, and immune cells has been largely investigated in the latest years; particularly, clinical and experimental evidences have proven that the main cytokines involved in the pathogenesis of inflammatory changes observed in RA play a significant role in systemic and local bone loss typical of this disease. TNF*α*, IL-1, and IL-6 blockade are not only able to prevent the structural joint damage, but also to prevent bone loss in RA. Whether the available biologic drugs against TNF*α*, IL-1, and IL-6 exert their effects on systemic and local bone loss through a direct mechanism on bone cell metabolism or indirectly by reducing local and systemic inflammation is still to be clearly defined. It has to be underlined that available data derives from short-term studies; thus, remains to be established if bone quality is affected with long-term use of these drugs. Further investigations into longer-term data are necessary to clearly define the potential risk and beneficial effects of biological drugs on bone tissue and to definitely assess the effect of these drugs on bone metabolism and on BMD and fracture risk in RA patients. 
